# An Interesting Case of Life-Threatening Hypercalcemia Secondary to Atypical Parathyroid Adenoma versus Parathyroid Carcinoma

**DOI:** 10.1155/2014/473814

**Published:** 2014-05-13

**Authors:** Ankur Mishra, David Newman

**Affiliations:** ^1^University of North Dakota, Apartment 402, 21 Broadway S, Fargo, ND 58103, USA; ^2^Sanford Health, Fargo, ND 58103, USA

## Abstract

*Context.* Severe hypercalcemia is a life-threatening condition. Atypical parathyroid adenoma and parathyroid carcinomas are uncommon causes which can be difficult to differentiate. *Objective.* We report a case of a 36-year-old male with very high serum calcium due to a possible atypical parathyroid adenoma versus parathyroid carcinoma. *Case Illustration.* A serum calcium level of 23.2 mg/dl was noted on admission. He was initially treated with IV hydration, pamidronate, and salmon calcitonin to lower his calcium levels. He also underwent a surgical en bloc resection of parathyroid mass. Pathology showed a mixed picture consistent with possible atypical adenoma versus parathyroid carcinoma. However, due to the possible involvement of the recurrent laryngeal nerve, parathyroid carcinoma was more likely. Also after operation the patient developed hungry bones syndrome and his calcium was replaced vigorously. He continues to be on calcium, vitamin D, and calcitriol supplementation. *Results.* A review of the literature was conducted to identify previous studies pertaining to parathyroid adenomas and parathyroid cancer. *Conclusion.* We thereby conclude that hypercalcemia requires very careful monitoring especially after operation. Also it can be very difficult to distinguish between atypical parathyroid adenomas and parathyroid carcinomas as in our case and no clear cut guidelines yet exist to differentiate the two based on histology.

## 1. Introduction

Acute primary hyperparathyroidism with/without hypercalcemic crisis is a rare but life-threatening emergency characterized by severe hypercalcemia and classical symptoms of calcium intoxication. This crisis most often occurs in cases of parathyroid adenoma or very rarely carcinoma.

We present this case of severe hypercalcemia to emphasize the severity of disease associated with parathyroid carcinoma with an emphasis on early management, diagnosis, and interventions to prevent any lifelong complications and any permanent organ dysfunction.

## 2. Case Report

This is a 36-year-old Caucasian male who presented with neck mass, muscle soreness, and joint pains. He noticed a progressively enlarging asymptomatic mass in the right lateral side of his neck adjacent to the thyroid cartilage for the past year. For the past few months or so he had noticed progressive decline in his ability to walk secondary to increasing muscle soreness and joint pains. He also complains of constipation and his last bowel movement was about a week ago. Notably he does have a sibling and aunt with thyroid issues, including goiter. There was no family history of stones or any other bone/kidney disease. He had been healthy previously with no other comorbidities.

**Figure 1:**
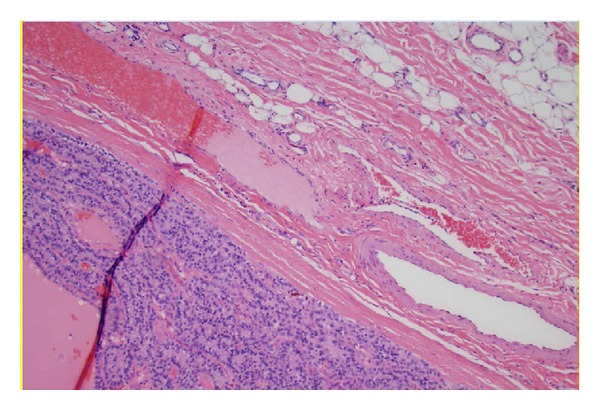
Parathyroid tumor capsule surrounding the soft tissue, 20x magnification.

**Figure 2:**
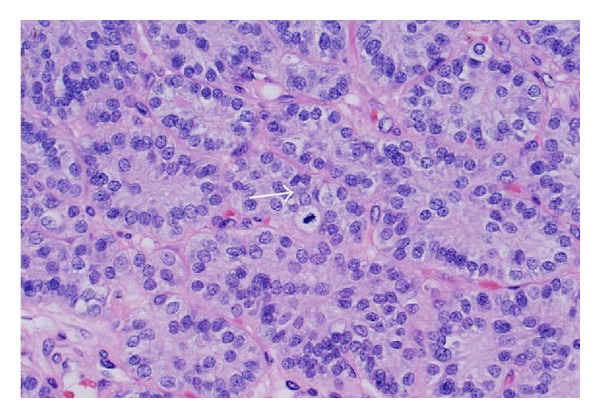
Parathyroid tumor cytology, arrow showing mitotic figure.

**Figure 3:**
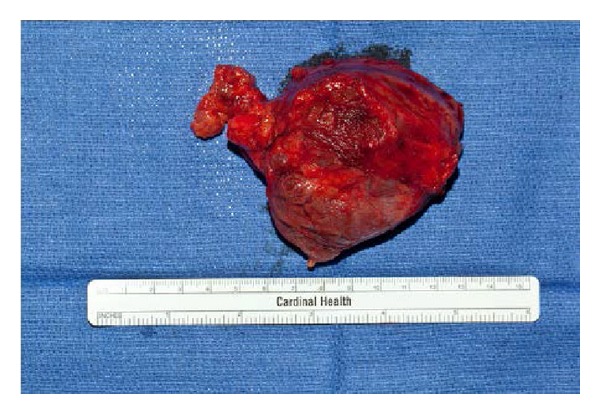
Gross pathologic specimen of the surgically resected parathyroid mass.

A physical examination revealed the following: stable vital signs; body temperature, 36°C; respiratory rate, 20 breaths/min; blood pressure, 122/68 mm Hg; heart rate, 90 beats/min, regular rhythm; and no pathological murmurs. His Ca on the date of admission was 23.2 mg/dL and albumin was 3.9 gm/dL. His PTH intact was 3342.5 pg/mL. His creatinine was elevated at 5 mg/dL, with the last known baseline of 1 mg/dL about a year back. Magnesium was 1.5 mg/dL and phosphorous was 3.4 mg/dL. Vitamin D 25 hydroxy and 1,25 dihydroxy were low at <14 ng/mL and 13 pg/mL, respectively. We did not do a 24 hr urine calcium as our suspicion for familial hypocalciuric hypercalcemia was low [1]. Renal ultrasound did not reveal any stones. There was no genetic analysis performed. Thyroid ultrasound showed that there was a large mass that appeared almost exophytic off of the right lobe of the thyroid. The patient was started on intravenous hydration (he received close to 10–12 L of normal saline in total before surgery), 1 dose of 90 mg pamidronate, and injectable salmon calcitonin 4 IU/Kg every 12 hours for 3 days. We also used cinacalcet (sensipar) to bring down calcium. Tc99 Sestamibi scan confirmed the findings of the thyroid ultrasound and it was recommended to obtain tissue diagnosis. Three days later after his calcium levels were stabilized and brought down to about 12 mg/dL he was taken to operating room and he underwent cervical exploration with right en bloc parathyroidectomy (without the violation of capsule and along with the tissues attached to it however the other right parathyroid was not identified) and thyroid lobectomy and his recurrent laryngeal nerve was sacrificed in the process as the mass was encompassing it (interestingly the patient did not have voice changes prior to surgery). The resected specimen weighed 101 grams and was 7.2 × 5.9 × 3.6 cm and was sent for pathology (Figure 3). There are only a few case reports with bigger giant parathyroid adenomas [2]. This was reviewed by the endocrine pathologist. The final report was initially supportive for atypical parathyroid adenoma. 29 lymph nodes were resected (along the right internal jugular vein and compartments 2, 3, 4, and 6 on the right side of neck) and were negative for any cancer. The lesion was encapsulated and no invasive growth was identified despite extensive histological sampling (Figure 1). The cells showed mild atypia with mild nuclear enlargement and pleomorphism. Mitotic figures were rare (Figure 2). No tumor necrosis was identified. However in the final report the pathologist also mentioned that this can still be considered as a parathyroid carcinoma if the recurrent laryngeal nerve was involved.

Post-op, his calcium, magnesium, and phosphate were closely followed due to the possibility of hungry bones syndrome. PTH intact came down to normal levels. Also his creatinine started trending downwards and he was having a very good urine output. His PTH intact had dropped to 12.3 pg/mL after surgery and was climbing up to about 108 pg/mL, 4 days post-op. The patient started developing perioral numbness and tingling in his extremities. Chvostek's sign was positive. His Ca dropped to 6.4 mg/dL. He required vigorous replacement of Ca with IV calcium gluconate up to 10 g of calcium a day along with oral Ca replacement of about 2-3 g q 6 hours. He was also started on calcitriol 1 mcg TID. The patient had developed hungry bones syndrome [3]. Interestingly, the patient's phosphorous always remained normal. He did have a mild degree of hypomagnesemia (with a Mg of 1.6–1.7 mg/dL on a few occasions) which was replaced adequately. We were getting labs every 6 hours for initial few days. After about 5 days of vigorous calcium replacement, he was finally discharged on oral calcium 3 g q 6 hours, vitamin D supplementations, and calcitriol 1 mcg TID. His calcium was 8.2 mg/dL on the day of discharge and it was followed every day after discharge in the endocrinology clinic. Creatinine on the day of discharge was down to 1.8 mg/dL as well and was down to 1.2 mg/dL about 2 months after discharge. His calcium level at that time was 9.7 mg/dL. Also he developed hoarseness of voice post-op as his right recurrent laryngeal nerve was sacrificed during surgery. He received an ENT referral and underwent a vocal cord surgery (thyroplasty).

## 3. Discussion

Different case series have reported serum calcium in the range of 9.9–18.4 mg/dL and 11.4–15.6 mg/dL [4, 5] in patients with parathyroid carcinomas. Spinelli et al. reported three cases of parathyroid carcinoma, one of them had serum calcium of 20.2 mg/dL [6]. Sheikh and Islam reported a case with serum Ca of 20.6 mg/dL [7]. Our patient had serum calcium of 23.2 mg/dL which is amongst the highest reported with parathyroid carcinomas/adenomas as per our knowledge right after Hauwe et al, which was due to an adenoma [8] and a couple of other cases due to PTHrP where calcium levels were 23.6 mg/dL [9].

Severe hypercalcemia is defined as serum calcium levels >14 mg/dL. Primary hyperparathyroidism is often associated with borderline or mild hypercalcemia (serum calcium concentration often below 11 mg/dL). Values above 13 mg/dL are unusual in primary hyperparathyroidism, although they do occur, and are more common in patients with malignancy-associated hypercalcemia [10].

Parathyroid adenomas are the single most common cause of primary hyperparathyroidism. The incidence of parathyroid adenoma is reported to be about 85%. These are common in the inferior glands and also may be found in the ectopic regions such as mediastinum, thyroid, and retroesophageal. The most frequent symptoms are weakness, fatigue, anorexia, nausea, vomiting, polydipsia and polyuria, loss of weight, dyspepsia, constipation, and headaches. Bone, joint, muscular pain, pathological fractures, and renal stones are also common when hyperparathyroid state is prolonged and severe. Recurrent severe pancreatitis, anemia, and peptic ulcer disease can also occur in the later stages. However, sometimes the presenting sign is a palpable neck mass, especially in case of parathyroid carcinoma, as in our patient.

Parathyroid crisis is a rare and serious complication of primary hyperparathyroidism and is characterized by extremely high circulating levels of parathyroid hormone and acute onset of severe hypercalcemia (calcium > 3.5 mmol/L or 14 mg/dL) with associated symptoms of multiple organ failure, such as metabolic encephalopathy, renal insufficiency, gastrointestinal symptoms, and cardiac arrhythmia [11]. As such the threshold for treatment and transfer to ICU for severe hypercalcemic patients should be low.

Atypical parathyroid adenoma can be a difficult diagnosis to reach. Hence, those tumors whose features are worrisome, but not diagnostic of malignancy, fall under the rubric of “atypical adenoma.” According to Seethala et al. [12], the presence of 2 or more of the following attributes will lead to this diagnosis: incomplete invasion of the capsule, fibrous bands, pronounced trabecular growth, mitotic activity greater than 1 per 10 high-power fields, and tumor necrosis (that is not fine-needle aspiration or infarct related). Also consistent with the diagnosis of atypical parathyroid adenoma is the low number of cells staining for Ki-67 and MIB-1 [13, 14].

Our patient had no invasion of the capsule and had rare mitotic figures. No tumor necrosis was seen. It thus was more supportive of atypical adenoma; however, the pathologist still felt that clinical correlation was necessary and if the laryngeal nerve was involved then it should be classified as a parathyroid carcinoma. And interestingly in our patient the recurrent laryngeal nerve was found to be going right through this mass and for the same reason it was sacrificed during the surgery. However a specimen of this nerve was not available to be seen under the microscope to look for invasion (we do not know if this was a problem with the surgical technique or it got damaged in transition to a higher care center for an expert opinion). So should this mass be considered as a parathyroid carcinoma as per the final pathology report? Possibly staining with the above-mentioned techniques could have led to further diagnostic clues in the setting of the unavailability of the nerve specimen for pathological involvement.

Initial therapy of severe hypercalcemia includes the simultaneous administration of saline, calcitonin, and a bisphosphonate. This also includes concurrent administration of zoledronic acid (4 mg IV over 15 minutes) or pamidronate (60 to 90 mg over two hours), preferably zoledronic acid, because it is superior to pamidronate in reversing hypercalcemia related to malignancy [15]. Hemodialysis can also be considered for calcium levels >18 mg/dL with neurological symptoms and a stable circulation [16]. Cinacalcet (sensipar), a calcimimetic drug, is available for the treatment of secondary hyperparathyroidism associated with renal failure, hypercalcemia in parathyroid cancer, and the treatment of severe hypercalcemia in patients with primary hyperparathyroidism unable to undergo parathyroidectomy [17–19]. In our patient we used it effectively as well. However it is not a medical equivalent of parathyroidectomy as it is not permanent and does not reduce BMD. We propose that this can be used as a bridge in symptomatic individuals who are awaiting surgery. Another agent, gallium nitrate, inhibits osteoclastic bone resorption. Gallium also inhibits PTH secretion from parathyroid cells in vitro. Unlike bisphosphonates, gallium appears to be effective in both PTHrP-mediated hypercalcemia and non-PTHrP-mediated hypercalcemia. Preliminary data from clinical trials indicated that it is more potent than etidronate, pamidronate, and calcitonin alone [20–22].

## 4. Surgical Management

Parathyroid exploration is the preferred management and en bloc parathyroidectomy (complete surgical resection with microscopically negative margins) is performed if a parathyroid carcinoma is suspected intraoperatively [23].

The distinction between atypical adenoma and carcinoma can be quite a difficult one, and one may need to consider clinical findings in addition to the histological appearance. There is currently no pathognomonic immunophenotype to define neoplasia. In our patient since the final consensus was that it should be categorized as parathyroid carcinoma based on the fact that his recurrent laryngeal nerve was invaded (as per the surgeon) and hence sacrificed intraoperatively, he will need to be followed up every 3 months for the first 6 months and every 6 months for the 1st year and then annually as an outpatient for any signs and symptoms of recurrence just like that in a parathyroid carcinoma.
